# Cathepsins and neurological diseases: a Mendelian randomization study

**DOI:** 10.3389/fnins.2024.1454369

**Published:** 2024-10-03

**Authors:** Haitao Sun, Qingqing Tang, Xue Yan, Wanying Xie, Yueshan Xu, Weimin Zhang

**Affiliations:** ^1^Changchun University of Chinese Medicine, Changchun, China; ^2^The Third Affiliated Hospital of Changchun University of Chinese Medicine, Changchun, China

**Keywords:** cathepsin, neurological diseases, Mendelian randomization, risk, genetic associations

## Abstract

**Background:**

The causal relationship between cathepsins and neurological diseases remains uncertain. To address this, we utilized a two-sample Mendelian randomization (MR) approach to assess the potential causal effect of cathepsins on the development of neurological diseases.

**Methods:**

This study conducted a two-sample two-way MR study using pooled data from published genome-wide association studies to evaluate the relationship between 10 cathepsins (B, D, E, F, G, H, L2, O, S, and Z) and 7 neurological diseases, which included ischemic stroke, cerebral hemorrhage, Alzheimer’s disease, Parkinson’s disease, multiple sclerosis, amyotrophic lateral sclerosis, and epilepsy. The analysis employed various methods such as inverse variance weighting (IVW), weighted median, MR Egger regression, MR pleiotropy residual sum and outlier, Cochran Q statistic, and leave-one-out analysis.

**Results:**

We found a causal relationship between cathepsins and neurological diseases, including Cathepsin B and Parkinson’s disease (IVW odds ratio (OR): 0.89, 95% confidence interval (CI): 0.83, 0.95, *p* = 0.001); Cathepsin D and Parkinson’s disease (OR: 0.80, 95%CI: 0.68, 0.95, *p* = 0.012); Cathepsin E and ischemic stroke (OR: 1.05, 95%CI: 1.01, 1.09, *p* = 0.015); Cathepsin O and ischemic stroke (OR: 1.05, 95%CI: 1.01, 1.10, *p* = 0.021). Reverse MR analyses revealed that multiple sclerosis and Cathepsin E (OR: 1.05, 95%CI: 1.01, 1.10, *p* = 0.030). There is currently no significant relationship has been found between other cathepsins and neurological diseases.

**Conclusion:**

Our study reveals a causal relationship between Cathepsins B, D, E, and O and neurological diseases, offering valuable insights for research aimed at improving the diagnosis and treatment of such conditions.

## 1 Introduction

Neurological diseases, which are prevalent chronic disabling conditions, can significantly impair cognitive function and are characterized by high rates of morbidity, disability, and mortality, especially in low- and middle-income countries. As the population ages, environmental pollution increases, lifestyles change, and life expectancy rises, the health burden of neurological diseases is expected to rise (Cadilhac and Mahal, 2024; Huang et al., 2023). Despite substantial investments in disease research, the intricate pathogenesis and prolonged prodromal stages of these diseases impede the progress of developing effective treatments and prevention strategies. Unfortunately, definitive diagnosis often occurs late in the disease progression, resulting in missed opportunities for optimal treatment. Therefore, there is an urgent need for advancements in disease diagnosis. Biomarkers present a promising option for diagnosing neurological diseases due to their wide applicability, non-invasiveness, easy accessibility, and quantifiable clinical value, offering significant research and application potential (Hansson, 2021).

Cathepsins are a type of lysosomal protease commonly expressed in tissue cells and classified based on their proteolytic mechanisms into serine proteases, cysteine proteases, and aspartic proteases, among others (Yadati et al., 2020). Previous studies have demonstrated that cathepsins act as endopeptidases in lysosomal vesicles of normal cells, playing a significant role in various physiological processes such as cell differentiation, apoptosis, intercellular signaling, and maintenance of cell homeostasis (Huertas and Lee, 2022). Studies have demonstrated that Cathepsin B plays a crucial role in initiating neurodegeneration-related cell death and inflammatory processes associated with traumatic brain injury and related neurological disorders (Ni et al., 2022; Nakanishi, 2020). Furthermore, Cathepsin C promotes the polarization of microglia towards the M1 phenotype, thereby exacerbating neuroinflammation through the activation of the Ca^2+^ dependent PKC/p38MAPK/NF-κB signaling pathway (Liu et al., 2019). It is worth noting that, Cathepsin D is responsible for degrading misfolded proteins and regulating the activities of various polypeptides, enzymes, and growth factors in conditions such as Parkinson’s disease and Alzheimer’s disease (Mijanovic et al., 2021; Kang et al., 2021). Several observational studies have demonstrated elevated levels of Cathepsins H, L, and S in patients with Alzheimer’s disease (Pišlar and Kos, 2014), alongside upregulated levels of Cathepsins B and D in individuals with Amyotrophic Lateral Sclerosis (Stoka et al., 2023). Additionally, upregulation of Cathepsin E has been observed in the context of chronic neuroinflammation and brain injury (Ni et al., 2015). Animal studies indicate that Cathepsin H may exacerbate neurodegenerative diseases by intensifying neuroinflammation and contributing to neuronal death (Fan et al., 2015). Cathepsins S, K, and V are primarily involved in the remodeling of the extracellular matrix, which is closely linked to the neurodegenerative process (Stoka et al., 2016). Furthermore, the expression of Cathepsins B, D, and L is upregulated in experimental stroke models (Huertas and Lee, 2022), while the expression of Cathepsins B, D, and X is increased in animal models of Parkinson’s disease (Yusufujiang et al., 2024). The findings indicate a potential association between cathepsins and the onset and progression of neurological diseases. Nonetheless, conventional observational studies may be limited by biases and methodological constraints, hindering the establishment of a definitive causal relationship.

Mendelian randomization (MR) is a widely utilized technique in genetic epidemiology that employs single nucleotide polymorphisms as genetic instrumental variables (IVs) and adheres to the principle of independent assortment to investigate the causal association between specific traits and particular disease outcomes (Smith and Ebrahim, 2004). Genetic variation genotypes are determined at conception, ensuring random allocation and not being affected by disease progression. In contrast to observational studies, MR minimizes confounding variables, circumvents biases related to environmental or lifestyle factors, and eliminates the impact of reverse causality, thus providing a more precise indication of causality (Larsson et al., 2023; Smith and Ebrahim, 2003). This study examines the causal relationship between cathepsins and neurological diseases by analyzing data from a thorough genome-wide association study (GWAS).

## 2 Methods

As a pooled study analysis of publicly available data from GWAS studies, all studies underwent review and approval by the respective institution’s ethics review board, and every participant was granted informed written consent. As a result, no further ethics approval or license was necessary.

### 2.1 Study design

**Figure 1:**
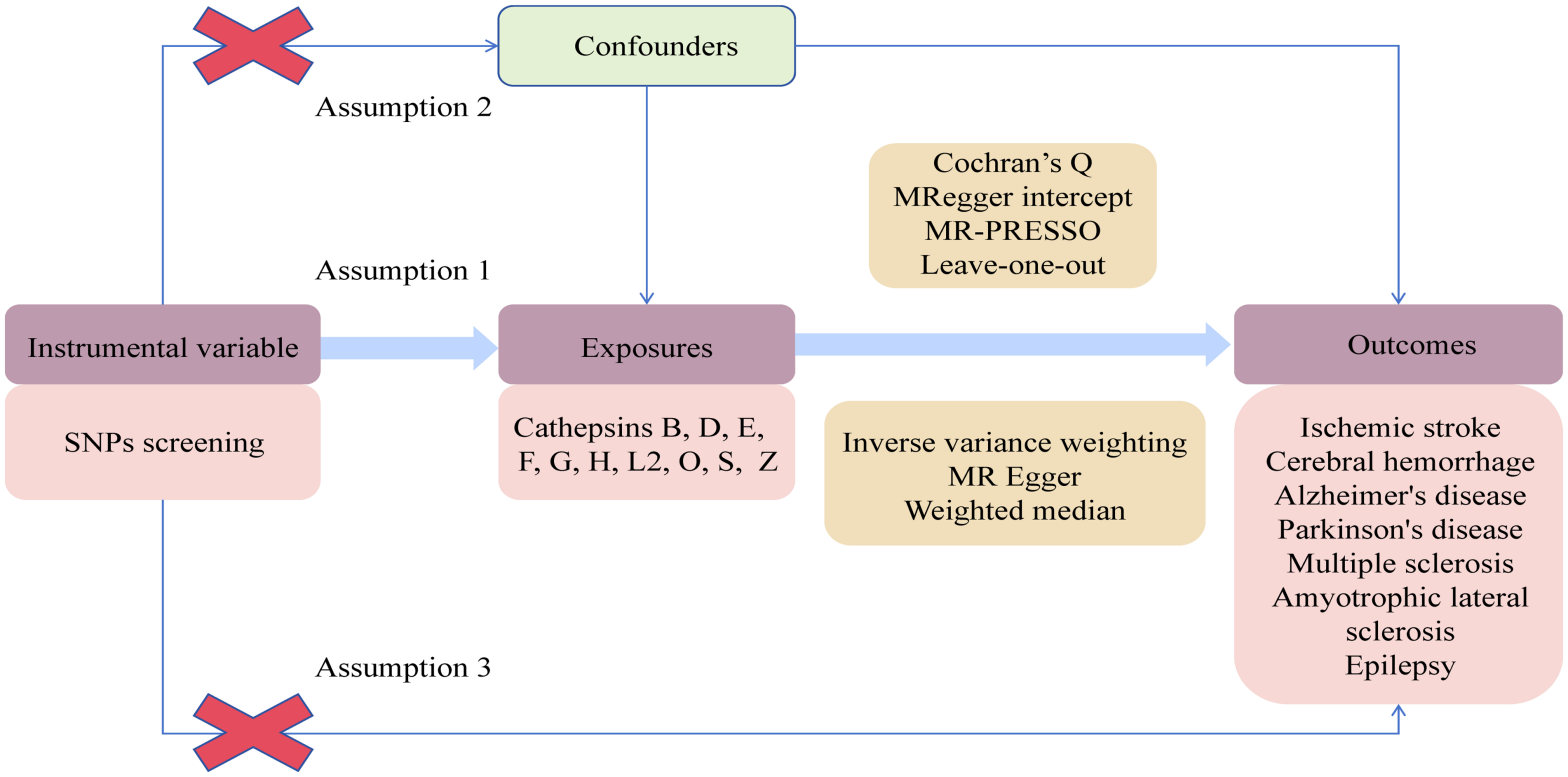
Flowchart of Mendelian randomization study on the causal relationship between cathepsins and neurological diseases.

A two-sample two-way MR experiment was conducted to assess the causal relationship between 10 cathepsins and 7 neurological diseases. The analysis relied on three key assumptions. Firstly, the genetic variation of the chosen IVs was reliably associated with the exposure factors. Secondly, these IVs were uncorrelated with potential confounding factors. Lastly, IVs only impact the outcome through the exposure factor and do not have any direct causal effect through other pathways. The experimental design is shown in Figure 1.

### 2.2 Source of IVs for cathepsins and neurological diseases

In this study, The 10 cathepsins (B, D, E, F, G, H, L2, O, S, and Z) datasets utilized in this study were derived from two extensive proteomics investigations (Folkersen et al., 2020; Folkersen et al., 2017). Statistics on neurological diseases are derived from a series of extensive GWAS conducted within European populations (Bellenguez et al., 2022; Nalls et al., 2019; International Multiple Sclerosis Genetics et al., 2019; van Rheenen et al., 2021; Sakaue et al., 2021). Specific details are provided in Supplementary Table S1. These samples were obtained from independent GWAS databases to ensure minimal overlap and bias. The data used in this study can be obtained from the Integrative Epidemiology Unit OpenGWAS project database.^1^

### 2.3 Study design and selection of IVs

We selected single nucleotide polymorphisms (SNPs) closely related to exposure factors (*p* < 5 × 10^−8^) as potential IVs (Supplementary Table S2). However, after the data for cathepsins other than Cathepsin D were subjected to such stringent criteria to eliminate linkage disequilibrium, we found ourselves lacking the necessary IVs (less than 3) to perform a comprehensive MR analysis. A significance threshold of *p* value less than 5 × 10^−6^ was used for the other 9 proteases to identify SNPs strongly correlated with the exposure under study (Wang et al., 2023; Zeng et al., 2024).

Subsequently, screening results underwent evaluation for linkage disequilibrium removal (LD r^2^ < 0.001, distance threshold >10,000 kb). To ensure the independence of IVs from potential confounders, we referred to the IEU database to eliminate SNPs linked to various risk factors such as smoking and alcohol consumption. Furthermore, SNPs strongly correlated with the outcome were also excluded. Finally, we calculated the *F* value for each SNP, identifying SNPs with F statistics less than 10 as weak IVs, which were then eliminated to mitigate bias (Papadimitriou et al., 2020).

### 2.4 Statistical analyses

Prior to analysis, we aligned the IVs of the exposure group with the effect alleles of the corresponding outcomes. This alignment helped in excluding ambiguous SNPs with inconsistent alleles and filtering out SNPs with ambiguous minor allele frequencies, thus ensuring the accuracy of our analysis. In result analysis, Inverse variance weighting (IVW) is the primary method used to assess overall causal effects (Burgess et al., 2015). IVW assumes that the instrumental variable affects the outcome only through the exposure factor. It combines effect estimates from multiple independent studies and yields statistically significant results when the *p* value is less than 0.05. Weighted median and MR Egger methods are utilized to enhance the consistency and robustness of our findings, helping to address the limitations of IVW estimates (Bowden et al., 2015; Bowden et al., 2016). It is important to note that the above two methods can be statistically inefficient and influenced by abnormal genetic variation.

Horizontal pleiotropy and heterogeneity can significantly influence the reliability of MR analysis outcomes. To enhance the credibility and strength of our research findings, we conducted essential sensitivity analyses. Heterogeneity was identified using the IVW method, with a Cochran Q-derived p value below 0.05 suggesting the presence of heterogeneity (Burgess et al., 2013). Given that a random effects model is utilized, the heterogeneities mentioned have minimal influence on the outcomes of our analysis. As a result, our conclusions remain primarily reliant on our main analysis method. The MR Egger method was utilized to calculate the intercept, where a significance level of *p* < 0.05 suggested the existence of horizontal pleiotropic effects (Verbanck et al., 2018). In cases where horizontal pleiotropy was observed in the results, we proceeded to employ the MR-Pleiotropy Residual Sum and Outlier test to identify and remove any outlier SNPs. Subsequently, a leave-one-out analysis was conducted to evaluate the impact of individual SNPs on the MR estimates, enhancing the credibility of our study. In this study, odds ratios (OR) and 95% confidence intervals (CI) were utilized to evaluate causality. An OR value less than 1 indicates the exposure is a protective factor for the outcome, while a value greater than 1 suggests it is a risk factor. MR analysis was conducted using the ‘TwoSample MR’ package in R software (version 4.2.3).

We conducted a Bayesian co-localization analysis of cathepsins that are significantly associated with neurological disease using the “coloc” R package. This analysis aims to determine whether these associations are attributable to a shared causal variant or if they are influenced by confounding due to linkage disequilibrium (Giambartolomei et al., 2014). In conjunction with previous research, we employed the default parameters p1 = 1E-4, p2 = 1E-4, and p12 = 1E-5 for this analysis (Chen et al., 2023). The posterior probabilities derived from the co-localization analysis support one of the following five hypotheses: PPH0, where SNPs are not associated with any traits; PPH1, where SNPs are associated with gene expression but not with neurological disease risk; PPH2, where SNPs are associated with neurological disease risk but not with gene expression; PPH3, where SNPs are related to both neurological disease risk and gene expression, but driven by different SNPs; and PPH4, where SNPs are related to both neurological disease risk and gene expression, driven by shared SNPs. A posterior probability greater than 0.75 for PPH4 is considered to be co-localization evidence with high support. PPH4 values less than 0.75 and greater than 0.5 were considered to have medium-support for co-localization evidence (Kia et al., 2021).

## 3 Results

Cathepsins were utilized as an exposure factor to assess its effect on the risk of neurological diseases, with the IVW method employed as the primary analytical technique. Univariate MR analysis results indicated a correlation between higher levels of Cathepsin B (IVW (OR): 0.89, 95% (CI): 0.83, 0.95, *p* = 0.001) and Cathepsin D (OR: 0.80, 95%CI: 0.68, 0.95, *p* = 0.012) with a decreased risk of Parkinson’s disease. Conversely, elevated levels of Cathepsin E (OR: 1.05, 95%CI: 1.01, 1.09, *p* = 0.015) and Cathepsin O (OR: 1.05, 95%CI: 1.01, 1.10, *p* = 0.021) were associated with an increased risk of ischemic stroke. No significant relationship was found between other Cathepsins and neurological diseases. Causal relationships were tested for heterogeneity and horizontal pleiotropy. The *p* values for both the Cochran Q test and the MR Egger intercept were above 0.05, indicating no evidence of heterogeneity or horizontal pleiotropy. Additionally, the leave-one-out analysis did not reveal any specific SNPs that significantly influenced the results, highlighting the robustness of these findings.

**Figure 2:**
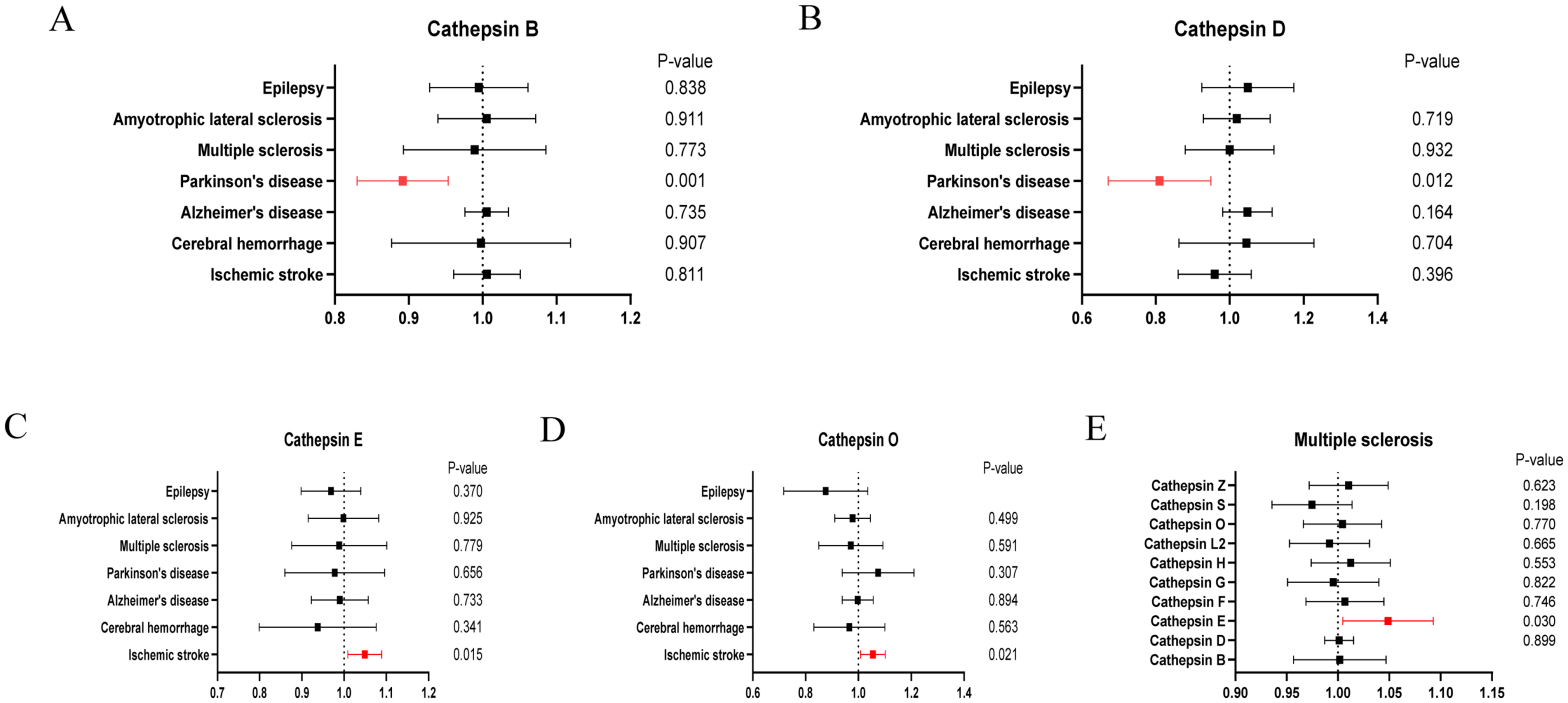
Forest plots of significant estimates of MR analyses. **(A)** Cathepsin B on neurological diseases. **(B)** Cathepsin D on neurological diseases. **(C)** Cathepsin E on neurological diseases. **(D)** Cathepsin O on neurological diseases. **(E)** Multiple sclerosis on cathepsins.

**Figure 3:**
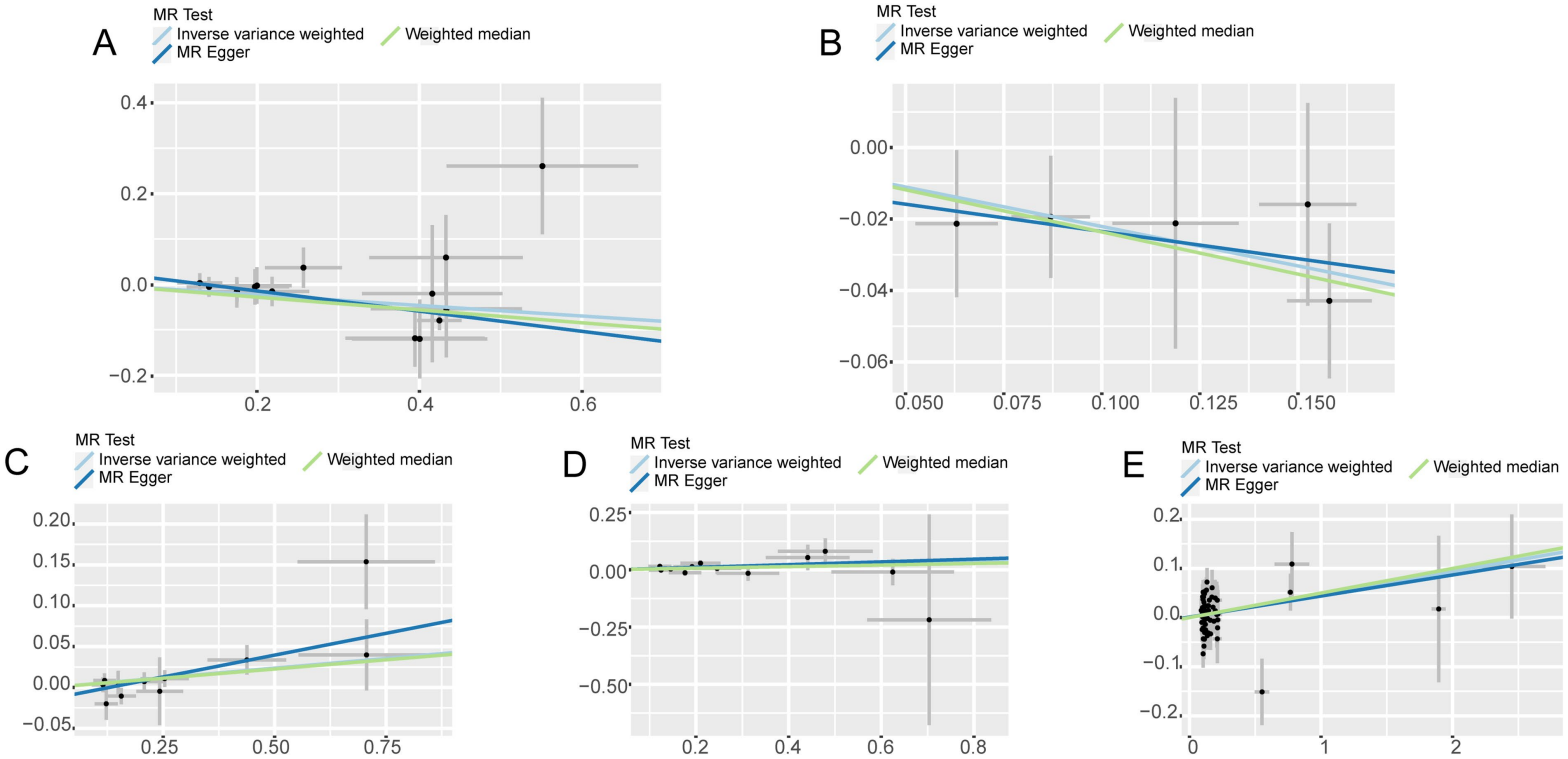
Scatter plot with forward causality in Mendelian randomization. **(A)** Cathepsin B on Parkinson’s disease. **(B)** Cathepsin D on Parkinson’s disease. **(C)** Cathepsin E on ischemic stroke. **(D)** Cathepsin O on ischemic stroke. **(E)** Multiple sclerosis on Cathepsin E.

**Figure 4:**
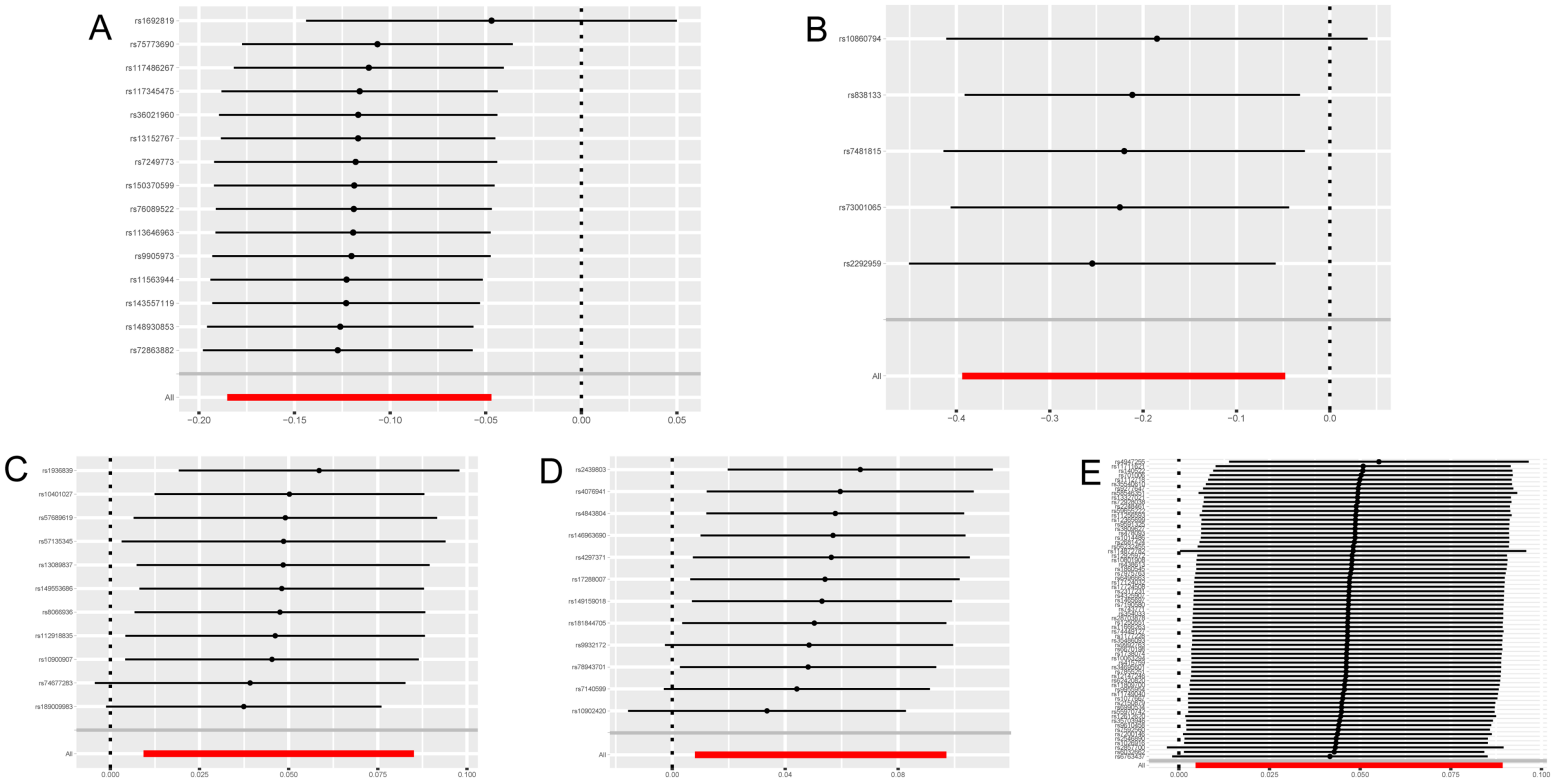
Leave-one-out sensitivity analysis with forward causality in Mendelian randomization. **(A)** Cathepsin B on Parkinson’s disease. **(B)** Cathepsin D on Parkinson’s disease. **(C)** Cathepsin E on ischemic stroke. **(D)** Cathepsin O on ischemic stroke. **(E)** Multiple sclerosis on Cathepsin E.

In addition to the primary analysis, reverse MR analyses were conducted to address potential reverse causality. The findings revealed a significant association between multiple sclerosis and elevated levels of Cathepsin E (OR: 1.05, 95%CI: 1.01, 1.10, *p* = 0.030), with no evidence of heterogeneity or horizontal pleiotropy (Supplementary Table S3). Detailed results are provided in Figures 2–4.

Co-localization analysis revealed that the PPH4 value for Cathepsin B (PPH4 = 0.705) demonstrated moderate evidence of co-localization with Parkinson’s disease (Supplementary Table S4). In contrast, the other cathepsins did not yield satisfactory results. Nevertheless, it is important to emphasize that negative results do not inherently undermine the findings derived from MR (Zuber et al., 2022).

## 4 Discussion

Nervous system diseases often present with an insidious onset and a protracted course, with causes and pathogenesis that remain incompletely understood, posing a significant threat to a patient’s health and quality of life. Cathepsin is known for its diverse biological functions, with past research suggesting potential links to neurological diseases through processes like protein decomposition, metabolic regulation, and inflammatory response. Through a two-way MR analysis of two samples, a potential association between cathepsins and neurological diseases was observed. Specifically, Cathepsin B and Cathepsin D were found to be protective against Parkinson’s disease. Conversely, Cathepsin E and Cathepsin O were found to be risk factors for ischemic stroke. Additionally, reverse MR indicated that multiple sclerosis is associated with reduced Cathepsin E levels.

Cathepsin B, a lysosomal hydrolase, plays a significant role in lysosomal storage disorders that share similar pathological features with Parkinson’s disease (Shachar et al., 2011). Various genetic studies on Parkinson’s disease have suggested that both common and rare variants in the CTSB gene, which encodes Cathepsin B, may increase the risk of developing PD (Chang et al., 2017; Milanowski et al., 2022). Previous research has shown that Cathepsin B is crucial for lysosomal degradation of *α*-synuclein, promoting the clearance of fibrillar α-synuclein in dopaminergic neurons and enhancing lysosomal function (Jones-Tabah et al., 2023). Additionally, Cathepsin B activity may also boost the function of glucocerebrosidase, encoded by the glucocerebrosidase gene. Notably, 5 to 10% of Parkinson’s disease patients exhibit mutations in the glucocerebrosidase gene (Schapira, 2015). This will result in reduced or absent glucocerebrosidase activity in macrophages, leading to the accumulation of glucocerebrosidase in lysosomes. This accumulation hinders *α*-synuclein degradation and promotes aggregation, ultimately resulting in the loss of cell function (Beutler, 2006). Maintaining normal levels of Cathepsin B could potentially have a beneficial impact on the treatment of Parkinson’s disease, while low levels of Cathepsin B may indicate a higher clinical risk for developing the disease.

Cathepsin D, a lysosomal proteolytic enzyme, is highly expressed in the brain. Its proteolysis plays a crucial role in the intracellular degradation of misfolded or non-functional proteins through autophagy or endocytosis, thereby maintaining the stability of nerve cells. This stability is particularly important for long-lived post-mitotic cells like neurons (Aufschnaiter et al., 2017). Research indicates that neurodegenerative diseases such as Parkinson’s disease feature protein aggregates of *α*-synuclein and amyloid precursors, which can result from genetic mutations or oxidative damage. Cathepsin D has shown effectiveness in degrading these key neuronal proteins (Kang et al., 2021). A study highlighted a significant interaction between the α-synuclein aggregation pathway and the role of lysosomal cathepsin. α-synuclein appears to directly inhibit the enzymatic function of cathepsin, leading to disruption in lysosomal transport. This disruption ultimately decreases the proteolytic activity of cathepsins, impacting α-synuclein clearance and potentially initiating a detrimental cycle of impaired α-synuclein degradation (Drobny et al., 2023). On the other hand, in cases where mutations in coding genes lead to reduced Cathepsin D enzyme activity in cell lysosomes, proteins, and other substances are not properly broken down and accumulate within lysosomes throughout the body. This accumulation, in the form of lipofuscin, results in neuronal damage (Drobny et al., 2023; Mijanovic et al., 2021). Therefore, it is essential to investigate the strong correlation between Cathepsin D and Parkinson’s disease, as it could potentially serve as a promising target for the treatment and prevention of this neurodegenerative disorder.

Cathepsin E is an intracellular aspartic protease that belongs to the pepsin superfamily. Previous studies have examined its expression in neurons and microglia to a certain extent (Chlabicz et al., 2011). Upregulation of Cathepsin E is predominantly associated with chronic neuroinflammation and brain injury. This upregulation may be attributed to the release of TNF-related apoptosis-inducing ligands by microglia surface proteolysis, which in turn modulates microglia activation via NF-κB, leading to accelerated plaque accumulation and deprivation of essential nutrients for cells (Ni et al., 2015). Regulation of intracellular levels of Cathepsin E in neurons occurs at both transcriptional and translational levels, and increased expression of Cathepsin E in damaged neurons and activated microglia in pathological brains can result in structural and functional alterations in neurons (Harada et al., 2017). Additionally, in the context of lipid metabolism and inflammation, cathepsins play a role in the formation of necrotic cores and foam cells, contributing to the development of atherosclerotic plaques through processes such as extracellular matrix remodeling and apoptosis (Ni et al., 2015). The formation of plaque plays a crucial role in the development of ischemic stroke. This process triggers an inflammatory response around the plaque, leading to gradual narrowing of the artery wall. If the thrombus dislodges, it can obstruct blood flow to the cerebral arteries, resulting in damage, necrosis, and softening of brain tissue (Endres et al., 2022). The upregulation of Cathepsin E may potentially hasten the progression of ischemic stroke. However, existing research on the correlation between Cathepsin E and ischemic stroke is limited, highlighting the necessity for more comprehensive studies to uncover the underlying mechanisms.

Cathepsin O is a type of cysteine protease. This type of protease is not only involved in intracellular protein degradation, but also has multiple functions, such as extracellular matrix protein degradation and signal transduction (Vidak et al., 2019). Throughout the progression of atherosclerosis, cysteine proteases play a significant role in the remodeling of extracellular matrix proteins, ultimately impacting the composition of elastin and collagen. Additionally, these proteases have a direct influence on the uptake, metabolism, and alteration of lipoproteins by macrophages, ultimately leading to the development of foam cells that are laden with lipid droplets (Wu et al., 2018). Upon the death of foam cells, plaques consisting of cellular debris, cholesterol, fibrous tissue proliferation, and calcium deposits are formed, ultimately leading to the development of atherosclerosis (Kim, 2021). As the disease progresses, damage to the vascular endothelium leads to the release of cathepsins, which then trigger inflammation and immune responses in peripheral blood vessels. This process leads to elastinolysis, recruitment of inflammatory cells, vascular apoptosis, and angiogenesis, all of which contribute to the deterioration of the intravascular environment and hasten the progression of ischemic stroke. It is important to note that there is a relative scarcity of research on Cathepsin O, and its precise role in ischemic stroke remains unclear. Therefore, further investigation into the relationship between the two is highly warranted.

Reverse MR analysis indicates a correlation between multiple sclerosis and increased levels of Cathepsin E. Multiple sclerosis is a progressive inflammatory demyelinating disease of the central nervous system. Current research suggests that the adaptive immune system, particularly T and B lymphocytes, plays a crucial role in driving the disease process (Jakimovski et al., 2024). This involvement includes atypical macrophages, antibody and complement activation, and immune-mediated apoptosis leading to damage to myelin and neurons. Cathepsins, as regulators of the immune system, have been implicated in immune imbalance (Shimizu et al., 2017). Studies have shown increased expression of cathepsins in both multiple sclerosis patients and animal models (Baeva et al., 2024; Shimizu et al., 2017). Inhibiting different cysteine cathepsins may have a protective effect. Another hypothesis suggests that neuroimmune communication by neutrophil activity may have a connection to Cathepsin E (Harada et al., 2019). Neutrophils can trigger mechanical allodynia, and neuropathic pain often manifests before neurological symptoms in individuals with multiple sclerosis. This leads to the hypothesis that cathepsins could play a role in immune processes during the early stages of disease progression. However, due to the current lack of relevant studies, the exact relationship between these factors remains unclear, highlighting the need for further research.

This study investigated the causal relationship between cathepsins and neurological diseases. The findings regarding Cathepsin B and its association with Parkinson’s disease align with previous MR studies (Yusufujiang et al., 2024), thereby reinforcing existing knowledge. Importantly, this research expands the disease scope of investigation beyond earlier studies. The study also found a causal relationship between Cathepsin D and Parkinson’s disease, as well as between Cathepsin E and both stroke and multiple sclerosis, and between Cathepsin O and stroke. These insights may enhance our understanding of cathepsin related neurological diseases and inform potential treatment strategies.

This study has several limitations. First, the GWAS data used in the study were limited to the European population, which may constrain the generalizability of our findings. To enhance the strength of the evidence, expanding the study population is necessary to assess the generalizability of these findings to other ethnic groups such as Asia and Africa. Second, due to database constraints, we relaxed the threshold for screening IVs to ensure an adequate number of IVs for the study. This may have implications for the statistical power of the study, necessitating caution when interpreting the results. Lastly, our study is grounded on the theoretical link between cathepsin and neurological diseases, which calls for more extensive research to elucidate the mechanism of action and clinical significance further.

## 5 Conclusion

In conclusion, our study identified a causal relationship between Cathepsins B, D, E, and O and neurological diseases. This relationship may be linked to the involvement of cathepsins in inflammatory response, neuronal protein clearance, and extracellular matrix remodeling. To enhance clinical diagnostic and therapeutic effectiveness, additional experiments are required to elucidate the underlying mechanisms.

## Data Availability

The original contributions presented in the study are included in the article/Supplementary material, further inquiries can be directed to the corresponding author.

## Footnotes

^1^ https://gwas.mrcieu.ac.uk
